# DSCANet: Integrating dual encoder and spatial cross-attention for polyp segmentation

**DOI:** 10.1371/journal.pone.0345515

**Published:** 2026-04-20

**Authors:** Jun Su, Tiantian Shi, Bogdan Adamyk

**Affiliations:** 1 School of Computer Science, Hubei University of Technology, Wuhan, China; 2 Aston Business School, Aston University, Birmingham, United Kingdom; Samsun University: Samsun Universitesi, TÜRKIYE

## Abstract

Colonoscopy is a crucial clinical procedure for detecting colorectal polyps, which are strongly associated with the development of colon cancer. This endoscopic technique plays a vital role in both cancer prevention and early diagnosis. Accurate and efficient polyp segmentation is critical for enhancing the diagnostic reliability and clinical utility of colonoscopy. However, achieving precise segmentation presents significant challenges, primarily due to the diversity of polyps in their size and shape, coupled with poorly defined boundaries between polyps and surrounding tissues. To address these challenges, we propose a novel segmentation network, named DSCANet, which is a dual-branch encoder-structured network designed to efficiently fuse body and edge features for high-precision medical image segmentation. DSCANet integrates four key modules: a dual-branch encoder, a spatial cross-attention (SCA) module, a bipolar fusion (BF) module, and a flexible axis-attention (FAA) module. The dual-branch encoder consists of separate body and edge encoders, which extract respective features independently. The SCA module bridges the semantic gap between the two encoders’ features. The BF module fuses the shallowest and deepest features, while the FAA module assists the decoder in extracting semantic information from high-level features. DSCANet achieved superior performance on multiple colorectal polyp segmentation datasets. The code is available at https://github.com/Shantdst/DSCANet.

## 1. Introduction

Colorectal cancer (CRC) is a leading gastrointestinal malignancy that predominantly develops from the malignant transformation of colorectal polyps. Given the high incidence of CRC and its significant preventability, it has become a central focus of research in cancer prevention and treatment [1]. Numerous studies have demonstrated that the development of CRC is closely associated with the cancerous transformation of adenomatous polyps. Therefore, early detection and timely resection of these lesions are crucial for preventing and treating CRC. Clinical practice confirms that endoscopic resection of adenomatous polyps can reduce the risk of CRC by up to 90\%, demonstrating the critical importance of early detection in CRC prevention and control. At present, colonoscopy is the preferred method for screening, diagnosing, and treating colorectal polyps. While the widespread use of this technique has reduced CRC incidence by approximately 30\%, it still has certain limitations.

Deep learning-based medical image segmentation has significantly advanced clinical diagnosis [2,3]. While convolutional neural networks (CNNs) remain predominant in current segmentation approaches [4,5], their limited receptive fields restrict them to local feature extraction, often failing to model long-range spatial dependencies. Transformers [6] mitigate this limitation through self-attention mechanisms, enabling global contextual modeling.

**Fig 1:**
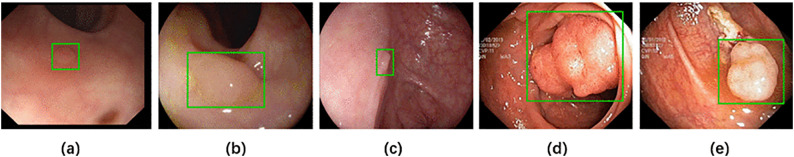
Some examples of typical polyps (a) and (b) indicate samples with blurred boundaries. (c) indicates a tiny polyp sample and (d) indicates a large polyp sample. (e) Indicates polyp samples with different colors.

In addition, polyp segmentation remains a challenging task, attributed to the variability in polyp size and shape, as well as poor target-to-background contrast ratio. As illustrated by the sample images of a polyp in Fig 1, the morphological diversity of polyps complicates their segmentation from the surrounding tissue. The substantial variability in size and the indistinct, often blurry boundaries further exacerbate the difficulty of polyp segmentation. To overcome these limitations, we developed an innovative multi-scale feature integration framework, termed DSCANet, optimized for polyp segmentation accuracy. The key contributions of this work include:

- We propose a hybrid network with a dual-branch encoder architecture, which is designed to separately extract edge and body features.
- We introduce the Spatial Cross-Attention (SCA) module to bridge the semantic gap between the edge and body branches before feature fusion, thereby enhancing segmentation accuracy.
- The model includes a two-stage fusion module that optimally combines shallow and deep feature representations.
- We integrate a lightweight Flexible Axis-Attention (FAA) layer into the decoder, achieving significant performance improvements with negligible computational overhead.

## 2. Related works

### 2.1. Traditional approaches in polyp segmentation

Polyp segmentation approaches primarily fall into two paradigms: conventional image processing techniques and machine learning-based methods. Conventional approaches employ methods such as thresholding [7], edge detection [8], and region-based segmentation [9] by utilizing low-level features (e.g., color, texture, and shape).

In contrast, machine learning-based methods demonstrate superior performance in extracting discriminative color and texture features for polyp segmentation. For example, Li et al. [10] enhanced polyp segmentation by projecting image features into higher-dimensional spaces via machine learning. Maghsoudi et al. [11] proposed a vectorization method that clusters pixels with similar features for efficient segmentation.

While these conventional methods [12] are valuable, they depend on handcrafted features and exhibit limited generalizability for colorectal polyp analysis. This is particularly evident in scenarios characterized by complex contours, significant morphological diversity, and low-contrast conditions, which consequently lead to limited precision in polyp localization and mucosal boundary differentiation.

### 2.2. Deep learning methods in polyp segmentation

In recent years, the rapid advancement of deep learning techniques has enabled convolutional neural networks (CNNs) to significantly contribute to polyp segmentation through their powerful multi-scale feature encoding capabilities [13]. A seminal architecture, U-Net [14], introduced by Ronneberger et al., established an encoder-decoder symmetry with skip connections. Building upon this, Zhou et al. [15] developed U-Net++, which incorporates dense skip connections to significantly enhance multi-scale feature fusion and effectively bridge the semantic gap across network hierarchies. Other U-Net variants, including Levit-U-Net [16], PDAtt-U-Net [17], ERD-U-Net [18], and ADS-U-Net [19], further incorporate attention mechanisms, pyramid pooling, and advanced feature aggregation to optimize the framework for polyp segmentation challenges.

In computer vision, Transformers are recognized for their superiority in extracting global information and capturing long-range dependencies, whereas CNNs excel at modeling local features. Consequently, a series of novel Transformer-CNN hybrid architectures have been developed for polyp segmentation to leverage the complementary strengths of both paradigms. For instance, TransFuse [20] employs a parallel branching structure to fuse CNN and Transformer features, while SwinENet [21] couples EfficientNet’s local feature extraction with the Swin Transformer’s long-range dependency modeling. This design preserves hierarchical feature representations across both global and local scales, thereby significantly enhancing segmentation robustness and accuracy. The MIA-Net framework [22] achieves multi-scale information fusion through the parallel processing of Transformer attention mechanisms and convolutional feature maps. By incorporating dedicated feature extraction and cross-modal fusion modules, MIA-Net effectively improves segmentation accuracy. HSNet [23] synergistically integrates CNN and Transformer architectures for comprehensive local-to-global feature representation. In contrast, ColonFormer [24] employs an efficient Transformer encoder for multiscale feature learning and uses a CNN-based decoder to enable hierarchical feature aggregation, thereby improving accuracy for small polyps.

To fully leverage the rich information in medical images and significantly enhance segmentation precision, we propose DSCANet, an advanced dual-encoder hybrid model for polyp segmentation. Our model is designed to address the semantic gap prior to feature fusion, thereby enhancing the integration of body and edge information. Furthermore, it overcomes the limitations of previous feature fusion techniques and effectively extracts multi-level and fine-grained features, demonstrating outstanding segmentation performance.

## 3. The proposed method

**Fig 2:**
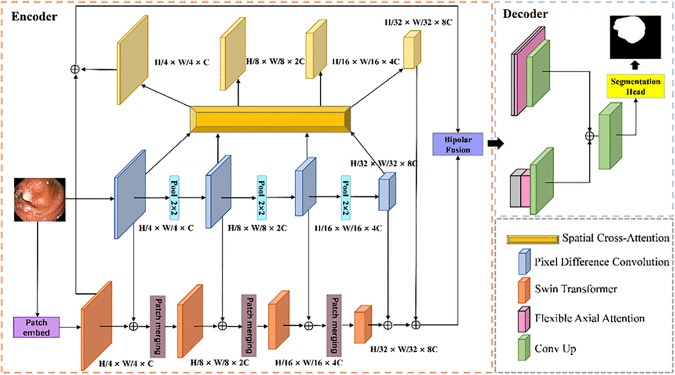
The structure of the proposed DSCANet.

The overall architecture of our proposed DSCANet is illustrated in Fig 2, which primarily consists of four key components: a dual-branch encoder, a Spatial Cross-Attention (SCA) module, a Bipolar Fusion (BF) module, and a Flexible Axis-Attention (FAA) module. The SCA module bridges the semantic gap between the two branching encoders prior to feature fusion, while the BF module is designed to effectively integrate high-level and low-level feature maps. The following subsections will provide a detailed exploration of each component.

### 3.1. Dual-branch encoder

The proposed dual-encoder architecture comprises two components: (1) a Swin Transformer-based body encoder [25] for global contextual modeling, and (2) a CNN-based edge encoder that employs Pixel Difference Convolution (PDC) [26] for local boundary delineation.

#### 3.1.1. Edge encoder.

To address the limitations of conventional medical image segmentation approaches in edge feature extraction, we introduce a dedicated boundary-aware module to enhance segmentation precision. As illustrated in Fig 2, the edge encoder employs a four-stage hierarchical architecture. Each stage integrates four Pixel Difference Convolution (PDC) blocks for multiscale feature extraction, with max-pooling operations between stages progressively reducing feature map dimensions to construct a pyramidal representation. The initial stage transforms the three-channel input into a C-dimensional feature space while performing 4 × spatial downsampling to ensure dimensional consistency with the body encoder.

The core component, the PDC module, is designed to overcome the limited edge perception of standard convolutions by incorporating gradient-aware operations. While vanilla convolution performs a weighted accumulation of pixel intensities within a kernel, PDC computes the weighted sum of pixel value differences, thereby explicitly capturing edge information. The mathematical formulations for vanilla convolution and PDC are provided in Equations 1 and 2, respectively. Each PDC block consists of a ReLU activation followed by depthwise and pointwise convolutional layers. Residual connections are incorporated to alleviate the vanishing gradient problem and facilitate more stable gradient flow through the deep network.


$$\begin{array}{c} y=f(x,\theta )=\sum \overset{k\times k}{\underset{i=\text{1}}{\mathrm{\backslash nolimits}}}\omega_{i}\cdot x_{i}\;(vanilla\;convolution) \end{array}$$ (1)

$$\begin{array}{c} y=f(\nabla x,\theta )=\sum {\mathrm{\backslash nolimits}}_{(x_{i},\overset{\prime }{\underset{i}{x}})\in P}\omega_{i}\cdot (x_{i}-\overset{\prime }{\underset{i}{x}})\;(PDC) \end{array}$$ (2)

Where $\;\omega_{i}$ denotes the weights within the k × k convolution kernel, while $x_{i}$ and $\overset{\prime }{\underset{i}{x}}$ represent the pixels covered by the kernel, and P refers to the assortment of pixel pairs selected within the kernel’s local coverage area. To enhance edge delineation further, we employ a supervised strategy that generates an edge map from the output features from each stage and calculates the difference between this edge map and the ground truth.

#### 3.1.2. Body encoder.

The body encoder utilizes the Swin Transformer architecture to generate high-level feature representations for global context modeling. Its shifted window mechanism enables efficient inter-region feature interaction, which has demonstrated superior performance in segmenting irregular organ structures. As indicated by the red blocks in Fig 2, the Swin Transformer encoder consists of four hierarchical stages. The initial stage employs a patch embedding layer to partition the input image into non-overlapping P × P patches. These patches are then linearly projected into a C-dimensional embedding space, incorporating positional encoding to preserve spatial information. The resulting sequence of token embeddings is processed by a series of Swin Transformer blocks.

In subsequent stages, patch merging operations downsample the feature maps while increasing their dimensionality, allowing the network to capture more complex features at larger receptive fields. Each stage is composed of multiple Swin Transformer blocks. Critically, these blocks are arranged in alternating pairs: one block employs a Window-based Multi-head Self-Attention (W-MSA) mechanism, while the subsequent block uses a Shifted Window-based Multi-head Self-Attention (SW-MSA) mechanism to enable cross-window communication. Each block comprises a Layer Normalization (LN) layer, a multi-head self-attention module (either W-MSA or SW-MSA), a Multilayer Perceptron (MLP), and residual connections skipping both the attention and MLP layers. The detailed computational procedures for these blocks are formalized in Equations (3) through (6).


$${\hat{z}}^{l}=W-MSA\left(LN\left(z^{l-\text{1}}\right)\right)+z^{l-\text{1}}\;$$ (3)

$$z^{l}=MLP\left(LN\left({\hat{z}}^{l}\right)\right)+{\hat{z}}^{l}\;$$ (4)

$${\hat{z}}^{l+\text{1}}=SW-MSA\left(LN\left(z^{l}\right)\right)+z^{l}\;$$ (5)

$$z^{l+\text{1}}=MLP\left(LN\left({\hat{z}}^{l+\text{1}}\right)\right)+{\hat{z}}^{l+\text{1}}\;$$ (6)

Patch merging is conducted between stages to downsample the feature map, thereby capturing essential contextual features. Adjacent 2 × 2 patches are combined into a single larger patch, effectively reducing the number and size of patches to minimize the loss of information. Patch merging reduces the feature scale by a downsampling ratio of two. Given an initial image size of H × W × 3, the resulting feature dimensions for each processing stage are denoted as H/4 × W/4 × C, H/8 × W/8 × 2C, H/16 × W/16 × 4C, and H/32 × W/32 × 8C, respectively.

### 3.2. Spatial cross-attention module (SCA)

The proposed Spatial Cross-Attention (SCA) module takes n feature levels from the edge encoder as input to generate enhanced representations. These refined features, particularly from the highest and lowest levels, are then connected to the corresponding stages of the body encoder, thereby providing richer semantic information.

**Fig 3:**
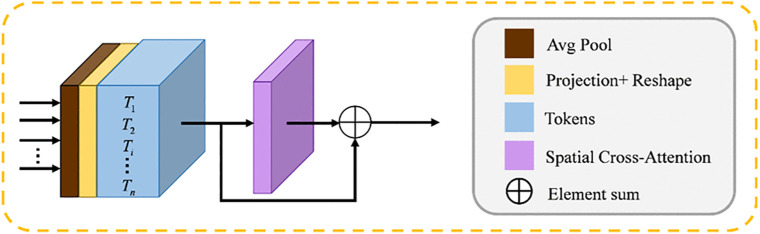
The SCA module.

As illustrated in Fig 3, the SCA module operates in two sequential phases:

1. **Multi-scale Feature Tokenization**: In this phase, multi-scale patch embedding modules process the input feature maps from the edge encoder to transform them into a sequence of tokenized representations.
2. **Cross-Attention Feature Refinement**: The subsequent phase applies the core Spatial Cross-Attention mechanism to these tokens, enabling the modeling of long-range dependencies across the spatial domain and refining the features based on global context.

Initially, multi-scale patches are extracted from four hierarchical stages of the edge encoder. Given four hierarchically scaled encoder stages $E_{i}\in R^{C_{u}\times H/{\text{2}}^{i-\text{1}}\times W/{\text{2}}^{i-\text{1}}}$ and corresponding patch sizes $\overset{s}{\underset{i}{P}}=\;P^{S}/{\text{2}}^{i-\text{1}}$ where i = 1, 2, 3, 4, we extract blocks using 2D average pooling with specified pooling and stride sizes $\overset{s}{\underset{i}{P}}$. Subsequently, we apply 1 × 1 deep convolutional mapping to the flattened 2D blocks:


$$T_{i}=DConv\text{1}D_{E_{i}}(Reshape(AvgPool\text{2}D_{E_{i}}(E_{i})))$$ (7)

where $T_{i}\in R^{P\times C_{i}}$, (i = 1, 2, 3, 4) denotes the i-th encoder stage of planarized patches. It is worth noting that the patch count P remains invariant throughout all processing stages $T_{i}$, allowing for the use of cross-attention among these tokens.

**Fig 4:**
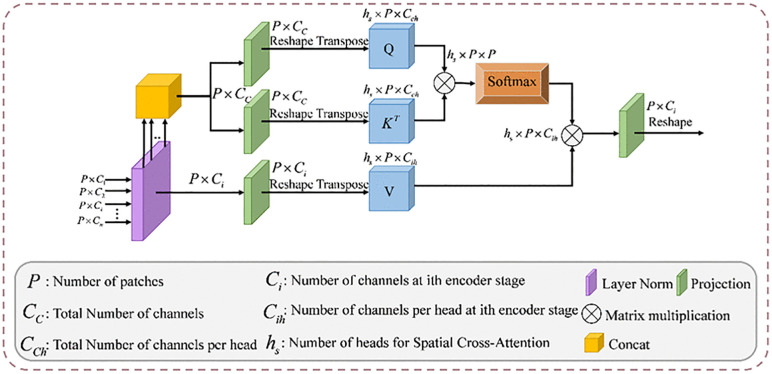
The spatial cross-attention mechanism.


The SCA mechanism is shown in Fig 4 Given the reshaped output $T_{i}\in R^{P\times C_{i}}$ (i = 1, 2,..., n), layer normalization is conducted followed by concatenation across the channel dimension. We use the connected tokens ${\bar{T}}_{c}$ as the query and key, along with each token ${\bar{T}}_{i}$ as the value. We use a 1 × 1 depth mapping for queries, keys, and values:

$$Q=DConv\text{1}D_{Q}({\bar{T}}_{c})$$ (8)

$$K=DConv\text{1}D_{K}({\bar{T}}_{c})$$ (9)

$$V_{i}=DConv\text{1}D_{V_{i}}({\bar{T}}_{i})$$ (10)

where $Q\in R^{P\times C_{c}}$, $K\in R^{P\times C_{c}}$ and $V_{i}\in R^{P\times C_{i}}$, are the predicted query, key, and value respectively. Then SCA can be represented as


$$SCA(Q,K,V_{i})=Softmax\left(\frac{{QK}^{T}}{\sqrt{d_{k}}}\right)V_{i}$$ (11)

in this context, Q, K, and $V_{i}$ denote the matrices of query, key, and value embeddings, respectively, and $\text{1}/\sqrt{d_{k}}$ is the scaling factor. In the multi-head configuration $\sqrt{d_{k}}=C_{c}/h_{c}$, where $h_{c}$ represents the total number of heads. The output of the SCA is then processed through deep convolution operation to generate SCA outputs. Layer normalization and GeLU activation are then applied to these SCA outputs. Finally, the n outputs from the SCA blocks are connected to the corresponding decoder sections via the upsampling layer, followed by the sequence of 1 × 1 convolution, batch normalization, and ReLU activation. Cross-attention differs from self-attention in that creates an attention map by fusing multiscale encoder features, rather than operating on each stage individually. This mechanism is utilized to capture long-range relationships across distinct encoder stages.

### 3.3. Bipolar fusion module(BF)

Currently, a major challenge lies in effectively fusing multi-level features from CNNs and Swin Transformers while maintaining feature consistency. Traditional approaches involve summing the features from CNN levels and integrating the associated Swin Transformer layers into the decoder, subsequently producing a segmentation map. However, this method makes it difficult to ensure feature consistency across different levels, leading to suboptimal performance. Consequently, we introduce a novel module named BF, which effectively addresses this issue by accepting the minimum ($P^{s}$) and maximum ($P^{l}$) levels as inputs and utilizing a cross-attention mechanism to combine multi-scale information.

Typically, shallower levels contain more precise localization information, whereas deeper levels contain more semantic content, which is ideally matched for the decoder. To efficiently fuse multi-scale features and reduce computational overhead, we selectively incorporate only the shallowest and deepest levels into the feature fusion process. In the proposed BF module, the class token plays a pivotal role by aggregating comprehensive information from the input features. These class tokens are generated through the Global Average Pooling (GAP) applied to each level’s features. The process for obtaining class tokens is described as follows:


$${ClS}^{s}=GAP\left(Norm\left(P^{s}\right)\right)$$

$${ClS}^{l}=GAP(Norm(P^{l}))$$ (12)

Where $CLS^{s}\in R^{\text{4}D^{\prime }\times \text{1}},\;CLS^{s}\in R^{D^{\prime }\times \text{1}}$. The class tokens are subsequently linked to the corresponding level embeddings before being passed to the transformer encoders. S transformer encoders are deployed at the smaller level, with L transformer encoders utilized at the larger level for global self-attention computation. Importantly, learnable position embeddings are incorporated into each token across both levels, integrating positional data into the learning process of the transformer encoders.

Upon transmitting the embedding information through the transformer encoder, the features of each level are integrated through the cross-attention module. Prior to fusion, the class tokens of the two levels are exchanged, meaning that the class tokens from one level are linked to those of the other level. Each newly generated embedding is then integrated via the module and subsequently reprojected to its corresponding level. Interactions between tokens from distinct levels allow class tokens to propagate extensive information across the levels.

**Fig 5:**
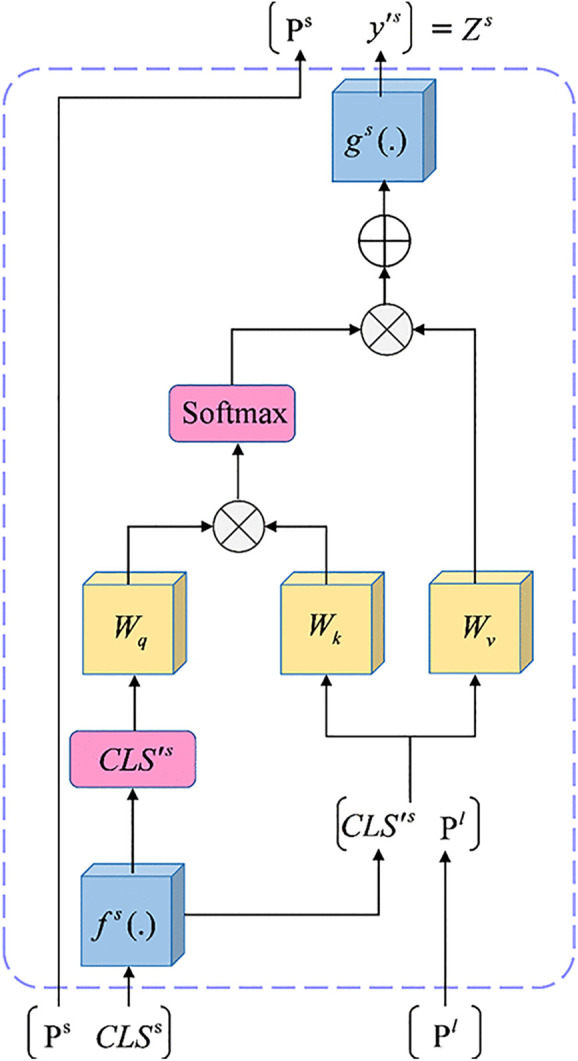
The BF module.


In particular, this interaction for smaller levels is illustrated in Fig 5 The output $f^{s}\;(.)$, obtained by initially projecting ${CLS}^{s}$ onto the dimension of $P^{l}$, is denoted as ${CLS}^{\prime s}$. ${CLS}^{\prime s}$. is linked to $P^{l}$. The key and value for the cross-attention calculation, while also function independently as a query. Given that only class tokens are queried, the cross-attention mechanism runs in linear time, producing the output $Z^{s}$, which can be expressed mathematically as:

$$y^{s}=f^{s}\left(CLS^{s}\right)+MCA\left(LN\left(\left[f^{s}\left(CLS^{s}\right)∥P^{l}\right]\right)\right)$$ (13)

$$Z^{s}=[P^{s}∥g^{s}(y^{s})]$$ (14)

### 3.4. Flexible axial-attention for decoder (FAA)

To enhance the decoder’s extraction of semantically rich high-level feature information, we analyze the advantages of employing an FAA mechanism applied to visual recognition. To accommodate computational constraints, operations are restricted to the height and width axes of the decoder feature map, implementing axial attention along these axes. This approach notably improves computational efficiency while replicating the self-attention mechanism.

Specifically, the axial attention mechanism in our study efficiently processes non-local contexts while achieving significant computational efficiencies. The method incorporates positional biases into its framework and effectively encodes remote interactions within the input feature maps. However, this approach performs best on large-scale datasets, as axial attention is more adept at learning positional biases among keys, queries, and values. For small-scale datasets, particularly in medical image segmentation, learning positional biases proves challenging. Consequently, we introduce an enhanced axial attention block to reduce the impact of positional bias on non-local context encoding. The attention mechanism applied to the width axis is formally expressed in Equation 15, with a similar formulation applying to the height dimension.


$${\text{y}}_{ij}=\sum_{w=\text{1}}^{W}softmax(\overset{T}{\underset{ij}{q}}k_{iw}+G_{Q}\overset{T}{\underset{ij}{q}}\overset{q}{\underset{iw}{r}}+G_{K}\overset{T}{\underset{iw}{k}}\overset{k}{\underset{iw}{r}})(G_{V\text{1}}v_{iw}+{\text{G}}_{V\text{2}}\overset{\nu }{\underset{iw}{r}})$$ (15)

**Fig 6:**
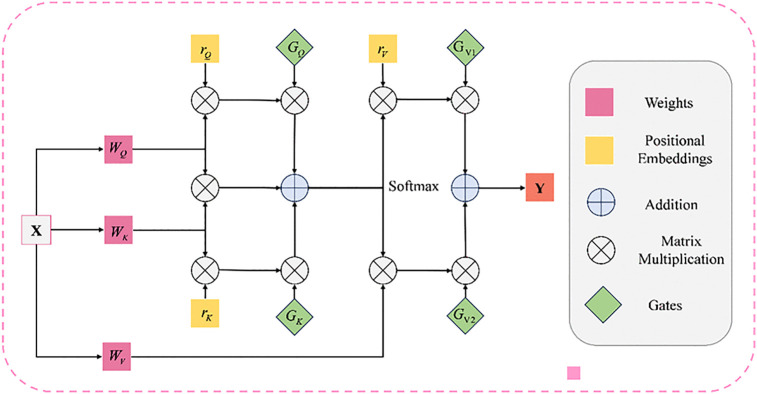
The FAA module.

The proposed framework incorporates an adjustable gating mechanism. The parameters $G_{Q,\;}G_{K,\;}G_{v\text{1},\;}G_{v\text{2},\;}$ are learnable and automatically updated, collectively forming the gating mechanism that controls the impact of learned relative position encodings on non-local context encodings. Typically, if a relative position encoding is precisely learned, the gating mechanism will prioritize it with a higher weight compared to that are not accurately learned. Fig 6 clearly illustrates the feedforward process of the gating mechanism in axial attention.

### 3.5. Loss function

To accommodate the two-branch structure of our network, we define a composite loss function consisting of two specialized components: $L_{Edge}$ and $L_{Body}$, each tailored to a specific branch.

#### 3.5.1. Edge supervision loss.

Since most edge detection samples yield negative results, we use an annotator-robust loss [27]. We implement this loss function across all hierarchical edge maps produced by the encoder’s multi-stage architecture. Specifically, for each spatial coordinate i in the j-th level edge detection output $\overset{j}{\underset{i}{y}}$, the loss is computed through a conditional evaluation framework defined as follows:


$$L_{Body}$$ (16)

In our formulation, $\overset{j}{\underset{i}{y}}$ denotes the predicted value of the i-th pixel in the j-th edge map. A predefined threshold γ determines the sample labeling: pixels annotated as positive but with confidence scores below γ are reclassified as negative. The dataset’s class imbalance is quantified by β, representing the proportion of negative samples. To balance the contributions of positive and negative samples, we introduce the weighting factor α=λ·(1−β), where λ is a hyperparameter. The hyperparameters for the edge loss are γ = 0.6, β = 0.8, λ = 2.0. The total edge loss $L_{Edge}$ is computed as the summation of per-pixel losses across all edge maps:


$$\begin{array}{c} L_{Edge}=\;\sum \overset{j}{\underset{i,jl_{i}}{\mathrm{\backslash nolimits}}} \end{array}$$ (17)

#### 3.5.2. Body supervision loss.

The inherent class imbalance in polyp image segmentation poses significant challenges. To mitigate this issue, we utilize a hybrid loss function that combines Binary Cross-Entropy Loss and Dice Loss, which effectively balances the learning between foreground and background regions.

The Binary Cross-Entropy Loss quantifies pixel-wise prediction errors through a logarithmic penalty, making it widely applicable for semantic segmentation tasks. Its formulation is given by:


$$\begin{array}{c} L_{BCE}=-\sum \overset{N}{\underset{m=\text{0}}{\mathrm{\backslash nolimits}}}\left[(\text{1}-{\hat{y}}_{i})log(\text{1}-y_{i})+{\hat{y}}_{i}log(y_{i})\right] \end{array}$$ (18)

In medical image analysis where foreground-background imbalance is prevalent, the Dice Loss function offers particular advantages by focusing on region-based overlap rather than pixel-level classification. The loss function is mathematically expressed as:


$$L_{Dice}=\text{1}-\text{2}\times \frac{\text{2}\sum_{i=\text{0}}^{N}y_{i}{\hat{y}}_{i}}{\sum_{i=\text{0}}^{N}\left(y_{i}+{\hat{y}}_{i}\right)}$$ (19)

Our hybrid loss function combines binary cross-entropy $L_{BCE}$ and Dice loss $L_{Dice}$ through linear combination:


$$L_{Body}=\lambda_{\text{1}}L_{BCE}+\lambda_{\text{2}}L_{Dice}$$ (20)

The overall loss function L integrates both edge-aware $L_{Edge}$ and region-based $L_{Body}$ components through a balancing hyperparameter:


$$L=L_{Body}+\omega \cdot L_{Edge}$$ (21)

We set the weight parameters λ₁, λ₂, and ω to 0.6, 0.4, and 0.3, respectively.

## 4 Experiments

### 4.1. Dataset details

We employ two benchmark medical image segmentation datasets, Kvasir-SEG [28], and CVC-ClinicDB [29] to evaluate our approach.

(1) The Kvasir-SEG dataset, focusing on pixel-level segmentation of colorectal polyps, comprises 1000 images of gastrointestinal polyps and their corresponding segmentation masks. The dataset is split with 80\% allocated to the training set and the remaining 20\% reserved for testing.
(2) The CVC-ClinicDB, used for segmenting lesion regions in colonoscopy images, includes 612 raw images and their corresponding segmentation masks. The data is divided into training and test sets in a 75\% to 25\% ratio.

To improve model robustness and prevent overfitting, we applied data augmentations including random horizontal/vertical flipping, random rotation, and color jittering in brightness, contrast, and saturation. These measures help the model generalize across variations in polyp shape, size, and visual appearance.

### 4.2. Implementation details

We implemented the proposed method using the PyTorch framework and conducted all experiments on an NVIDIA GeForce RTX 4060 8G GPU. To enhance computational efficiency, we resized all training, testing, and validation images to 224 × 224. The proposed model is trained using the Adam optimizer with a weight decay of 0.01 and an initial learning rate of 0.01. The learning rate is scheduled by the ReduceLROnPlateau algorithm with patience = 5, factor = 0.5, and min_lr = 1e-6. For comparison, we also conducted experiments using the SGD optimizer with an initial learning rate of 0.01 under the same scheduling settings. The batch size was set to 16 for both the Kvasir-Seg and CVC datasets.

All experiments were conducted using the aforementioned datasets, with Dice loss as the primary loss function. Performance was evaluated using the Dice Similarity Coefficient (DSC), mean Intersection over Union (mIoU), Precision, Recall, and F1 Score(F1). We trained both the proposed model and the comparison models from scratch for 200 epochs to ensure optimal performance on each dataset.

Below are the mathematical formulations for each of these metrics.


$$DSC=\frac{\text{2}\cdot TP}{\text{2}\cdot TP+FP+FN}\;$$ (22)

$$IoU=\frac{TP}{TP+FP+FN}$$ (23)

$$Precision=\frac{TP}{TP+FP}\;$$ (24)

$$Recall=\frac{TP}{TP+FN}\;$$ (25)

$$F\text{1}=\text{2}*\frac{Precision*Recall}{Precision+Recall}={\text{2}}^{*}\frac{TP}{TP+FP+TP+FN\;}$$ (26)

In this context, TP, TN, FP, and FN denote True Positives, True Negatives, False Positives, and False Negatives, respectively. These metrics are essential statistical measures derived from the comparison of predicted categories with actual categories.

### 4.3. Comparative experiment

To rigorously evaluate DSCANet’s performance advantages, we compare it with eight classic medical image segmentation models such as U-Net [14], ResUnet++ [30], MultiResUnet [31], R2U-net [32], VNet [33], DoubleU-Net [34], TransUNet [35], and BEFUnet [36].

#### 4.3.1. Results of Kvasir-SEG.

Table Table 1 presents a comparison between our proposed DSCANet and previous state-of-the-art (SOTA) methods on the Kvasir-SEG dataset. The experimental results indicate that the proposed method demonstrates exceptional segmentation accuracies, with scores of 95.28\% (DSC) and 90.98\% (mIoU). Compared to U-Net, its variants, and BEFUnet (which similarly employs a dual-encoder structure), our method demonstrates significant improvements in DSC evaluation metrics. Furthermore, DSCANet outperforms all competing methods in Recall. Notably, DSCANet surpasses both CNN-based methods (which rely solely on local information) and transformer-based approaches, demonstrating superior feature representation and edge prediction capabilities.

**Table 1:** Experimental results on the Kvasir-SEG dataset.

Model	DSC (%)	IoU (%)	Precision (%)	Recall (%)	F1 (%)
U-Net	92.23	85.71	91.87	87.86	86.73
ResUNet++	94.35	89.36	93.31	94.27	94.29
MultiResUnet	91.93	85.32	91.49	91.98	91,72
R2U-net	89.36	81.05	89.79	88.69	90.32
VNet	90.10	82.34	90.86	89.73	88.59
DoubleU-Net	94.51	89.63	94.52	95.39	92.16
TransUNet	94.47	89.58	93.36	95.12	93.71
BEFUnet	93.31	87.65	92.81	94.35	94.27
DSCANet	**95.28**	**90.98**	**95.64**	**95.85**	**94.46**

**Fig 7:**
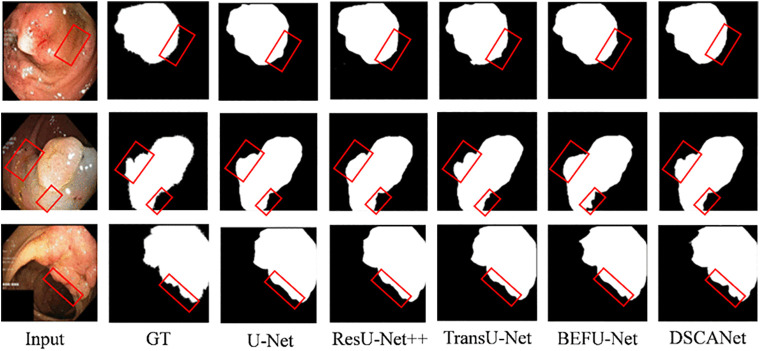
Visual illustration of the proposed method applied to the Kvasir-SEG dataset. The wrong target polyp is shown with a red outline.

Fig 7 compares visualized segmentation results obtained by several representative methods and our approach on the Kvasir-SEG dataset. The results highlight the limitations of existing methods in segmenting polyp edges, whereas our method effectively extracts edge features through its dedicated edge encoder, delivering superior segmentation performance.

#### 4.3.2. Results of CVC-ClinicDB.

Through experimental comparison of DSCANet with classical models on the CVC-ClinicDB dataset, our method maintains superior performance. As shown in Table 2, DSCANet outperforms all competing methods across all metrics, achieving segmentation accuracies of 95.45\% (DSC) and 91.28\% (mIoU), demonstrating balanced segmentation capability. The Precision and Recall metrics (95.86\% and 96.29\%, respectively) are 0.67 and 0.51 percentage points higher than those of DoubleU-Net, which is renowned for its excellent segmentation performance.

**Table 2:** Experimental results on the CVC-ClinicDB.

Model	DSC (%)	IoU (%)	Precision (%)	Recall (%)	F1 (%)
U-Net	92.53	86.16	94.06	88.43	84.18
ResUNet++	94.25	89.15	93.74	91.16	94.64
MultiResUnet	93.03	87.18	91.67	92.25	92.55
R2U-net	88.31	79.69	88.13	89.84	87.79
VNet	88.57	79.72	87.93	90.45	89.56
DoubleU-Net	94.63	89.96	95.19	95.78	93.63
TransUNet	91.92	85.23	91.06	92.31	93.47
BEFUnet	92.58	86.26	92.43	93.16	93.85
DSCANet	**95.45**	**91.28**	**95.86**	**96.29**	**95.62**

**Fig 8:**
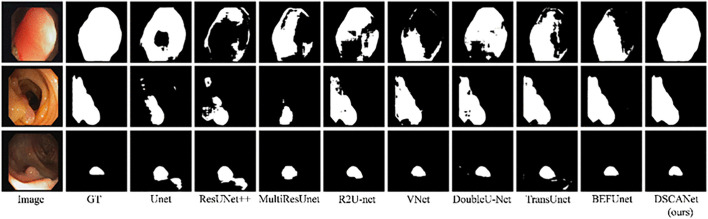
Visual illustration of the proposed applied to the CVC-ClinicDB dataset.

Fig 8 presents a visual comparison of segmentation results from multiple models on the CVC-ClinicDB dataset. Notably, our method produces results that more closely match the ground truth compared to other approaches. Evaluation across all three datasets demonstrates that DSCANet delivers comprehensive and robust segmentation performance, consistently achieving improved accuracy across diverse datasets. Specifically, DSCANet addresses the limitations of previous methods by effectively extracting and fusing both main body and edge features through multi-scale integration, enabling high-accuracy segmentation.

### 4.4. Qualitative analysis

Tables 1 and 2 presents the segmentation results of different models on the Kvasir-SEG and CVC-ClinicDB datasets. The results demonstrate that our method significantly outperforms the classical U-Net model, achieving Dice scores of 95.28\% and 95.45\% on the Kvasir-SEG and CVC-ClinicDB datasets, respectively. Among the compared methods, DoubleU-Net and TransUNet show outstanding segmentation performance on the Kvasir-SEG dataset. Compared to DoubleU-Net, our method improves the Dice score by 0.77 percentage points, mIoU by 1.35 percentage points, and Recall by 0.46 percentage points. Similarly, DoubleU-Net and ResUNet achieve excellent segmentation results on the CVC-ClinicDB dataset. Our method attains mIoU scores that are 1.32 and 2.13 percentage points higher than those of DoubleU-Net and ResUNet, respectively. Additionally, our method achieves the highest F1 scores on both datasets, confirming the superior overall performance of DSCANet.

### 4.5. Analysis of computational complexity

We evaluated computational efficiency along two primary dimensions: the number of parameters (Params), floating-point operations (FLOPs) and Inference Time. As summarized in Table 3, TransUNet has the largest parameter count (105.37M), indicating the highest memory demand among all competitors. Meanwhile, DoubleU-Net exhibits the highest computational cost at 130.93 GFLOPs, making it the least efficient model in terms of inference complexity. In contrast, our DSCANet requires only31.25M parameters and 58.63 GFLOPs and 0.0442 Inference Time significantly lower than most counterparts. This demonstrates that DSCANet maintains competitive performance while achieving superior computational efficiency and a reduced memory footprint.

**Table 3:** Computational complexity comparison of the evaluated models in terms of FLOPs and Params, with an input resolution of 224 × 224. The most efficient results for each column are shown in bold.

Method	FLOPs (G)	Params (M)	Inference Time(s)
U-Net	**30.27**	**7.88**	**0.0093**
R2U-Net++	65.4	16.12	0.0357
ResUnet++	102.13	39.49	0.0489
DoubleU-Net	130.93	44.34	0.0824
TransUNet	44.84	105.37	0.1115
Ours	58.63	31.25	0.0442

### 4.6. Ablation study

#### 4.6.1. Module ablation study.

**Fig 9:**
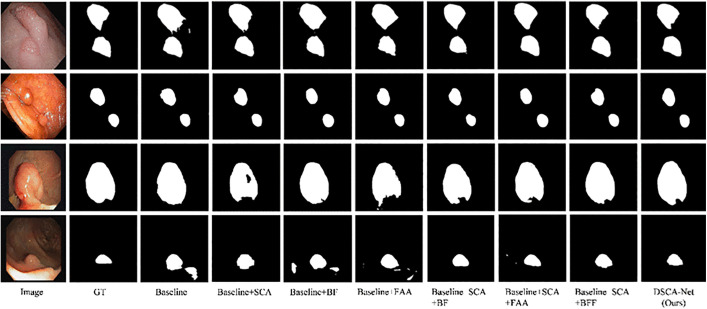
Visual illustration of module ablation study.

To validate the effectiveness of the key components in DSCANet, we conducted a comprehensive series of ablation experiments on the Kvasir-SEG and CVC-ClinicDB datasets. The baseline model consists of a two-branch encoder and decoder. We systematically enhanced the baseline by sequentially integrating the SCA, BF, and FAA modules, evaluating six distinct combinations: baseline + SCA, baseline + BF, baseline + FAA, baseline + SCA + BF, baseline + SCA + FAA, and baseline + BF + FAA. As demonstrated in Tables 4 and 5, each module contributes significantly to improving the baseline model’s performance on the Kvasir-SEG and CVC-ClinicDB datasets. dataset. The visualization results are presented in Fig 9.

**Table 4:** The module ablation experiments results on the Kvasir-SEG dataset.

Model	Setting	DSC (%)	IoU (%)	Precision (%)	Recall (%)
M1	Baseline	91.52	86.71	87.66	91.60
M2	Baseline+SCA	93.86	89.27	89.79	94.15
M3	Baseline+ BF	92.61	88.54	88.93	93.27
M4	Baseline+FAA	93.56	88.85	88.52	93.31
M5	Baseline+SCA + BF	94.49	90.31	94.18	94.68
M6	Baseline+SCA + FAA	94.85	90.72	94.37	95.22
M7	Baseline +BF + FAA	93.77	90.13	93.75	93.91
Ours	DSCANet	**95.28**	**90.98**	**95.64**	**95.85**

**Table 5:** The module ablation experiments results on the CVC-ClinicDB dataset.

Model	Setting	DSC (%)	IoU (%)	Precision (%)	Recall (%)
M1	Baseline	91.84	88.75	89.23	91.77
M2	Baseline+SCA	93.62	90.23	92.94	93.05
M3	Baseline+ BF	92.58	89.65	91.61	92.39
M4	Baseline+FAA	92.72	89.86	92.13	92.62
M5	Baseline+SCA + BF	93.89	90.68	93.17	93.88
M6	Baseline+SCA + FAA	95.14	90.95	94.48	94.51
M7	Baseline +BF + FAA	94.08	90.79	93.59	94.26
Ours	DSCANet	**95.45**	**91.28**	**95.86**	**96.29**

**Fig 10:**
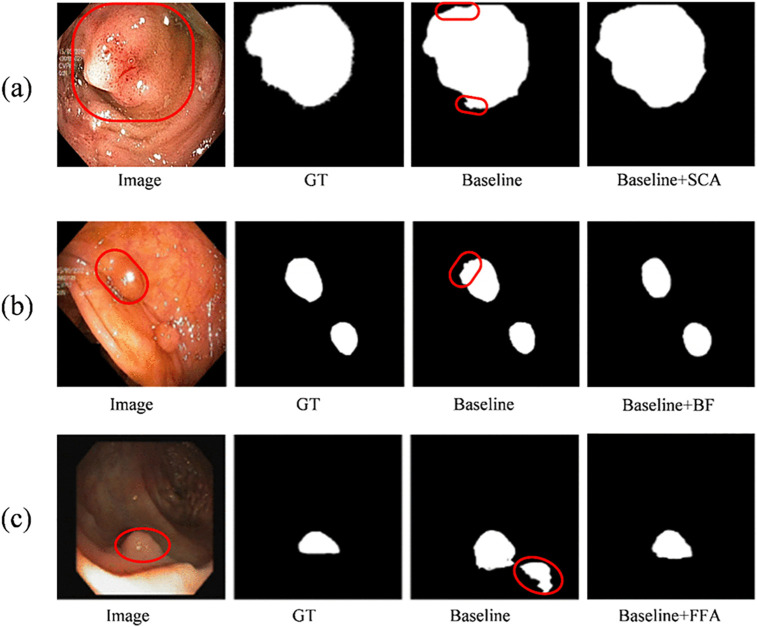
The detailed visual results of the module ablation experiments. The wrong target polyp is shown with a red outline.

**SCA Module Influence:** Model M2 (enhanced with SCA) achieves significant improvements in segmentation accuracy on both the Kvasir-SEG and CVC-ClinicDB datasets. The Dice scores improve by 2.34 percentage points and 1.78 percentage points, respectively, compared to the baseline model. Furthermore, SCA enhances all evaluated metrics, including mIoU, Precision, and Recall. The SCA module effectively bridges the semantic gap between the two encoders and facilitates multi-scale feature fusion, thereby significantly improving the model’s overall segmentation performance. As shown in Fig 10a, the SCA module markedly improves the baseline model’s boundary detection capability for large polyps, resulting in enhanced segmentation accuracy.

**BF Module Influence:** The proposed BF module effectively integrates lowest- and highest-level features, addressing the limitation of inadequate feature extraction in prior methods. When integrated with the baseline model on the Kvasir-SEG dataset, BF improves the DSC metric by 1.09 percentage points. Furthermore, when combined with SCA and FAA modules on the CVC-ClinicDB dataset, BF increases mIoU by 1.93 and 2.04 percentage points, respectively. As shown in Fig 10b, the baseline model is susceptible to noise interference, particularly in small polyp segmentation, resulting in reduced accuracy. By contrast, BF incorporation significantly enhances model robustness and improves detection performance for small polyps.

**FAA Module Influence:** The FAA component, enhances the decoder’s ability to extract high-level semantic features. Notably, the baseline+SCA + FAA combination achieves the second-best performance on Kvasir-SEG (after DSCANet), with a DSC of 94.85\%, mIoU of 90.72\%, Precision of 94.37\%, and the Recall of 95.22\% - representing significant improvements over the baseline. Comparative analysis shows that removing FAA from DSCANet reduces Precision by 1.46 and 2.69 percentage points on Kvasir-SEG and CVC-ClinicDB, respectively. Fig 10c demonstrates that while the baseline model struggles with noise artifacts in dark backgrounds, FAA integration dramatically improves robustness and segmentation precision.

#### 4.6.2. Loss function ablation study.

To validate the efficacy of the proposed body encoder loss strategy, we conducted comprehensive ablation studies while keeping the edge encoder’s loss function fixed. We evaluated the body encoder using both Binary Cross-Entropy (BCE) Loss and Dice Loss (DL) on the Kvasir-SEG and CVC-ClinicDB datasets. The quantitative results are presented in Table 6.

**Table 6:** Loss ablation experiments results on the Kvasir-SEG and CVC-ClinicDB datasets.

Loss strategy	Kvasir-SEG			
	DSC (%)	IoU (%)	Precision (%)	Recall (%)
Binary Cross-Entropy Loss	93.86	89.45	94.19	93.72
Dice Loss	94.53	90.17	94.36	95.09
Ours	**95.28**	**90.97**	**95.64**	**95.87**
	**CVC-ClinicDB**			
Binary Cross-Entropy Loss	94.33	90.52	94.28	94.30
Dice Loss	94.42	90.44	93.86	95.48
Ours	**95.45**	**91.28**	**95.86**	**96.29**

When employing Binary Cross-Entropy (BCE) loss alone for the body encoder, the model demonstrates significantly inferior segmentation performance compared to our hybrid loss strategy. Although BCE is widely adopted in semantic segmentation tasks, it suffers from a critical limitation: in scenarios with severe class imbalance (where foreground pixels are substantially outnumbered by background pixels), the background components dominate the loss function, leading to model bias toward background prediction and suboptimal performance.

This limitation is particularly evident in polyp segmentation, where polyp sizes exhibit considerable variation. While BCE achieves satisfactory results for larger polyps, its performance deteriorates significantly for smaller polyps. To address this challenge, we incorporate DL, which effectively mitigates the impact of foreground-background imbalance and enables more accurate segmentation of smaller polyps.

Notably, when employing either BCE or DL alone as the body encoder’s loss function, the model exhibits slower convergence compared to their combined usage. Moreover, within limited training epochs, our proposed hybrid loss strategy achieves superior performance, outperforming the standalone DL approach by 0.75 and 0.78 percentage points on the Kvasir-SEG and CVC-ClinicDB datasets, respectively, as measured by Dice scores.

**Fig 11:**
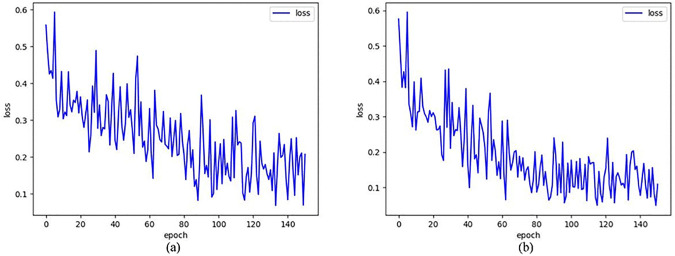
Training loss curve of different loss strategies on the Kvasir dataset. (a) Training loss curve of Binary Cross-Entropy Loss. (b) Training loss curve of combination of BCE and DL.

Our proposed hybrid loss function, which combines weighted BCE and DL, effectively handles the highly imbalanced foreground-background ratios in polyp segmentation. As shown in Fig 11, the hybrid loss strategy demonstrates significantly faster convergence compared to using BCE alone, while maintaining stable optimization behavior throughout training.

## 5. Conclusions

In this study, we propose DSCANet, a novel dual-branch encoder architecture designed for efficient multi-scale feature fusion. Our framework comprises three key components: A SCA module that bridges the body and edge encoders, effectively minimizing semantic gaps during feature fusion while enhancing feature integration; A BF module that hierarchically connects both the shallowest and deepest features to combine multi-resolution information; An FAA layer in the decoder that extracts high-level semantic features with computational efficiency. Extensive experiments on three public datasets demonstrate that DSCANet significantly outperforms state-of-the-art methods across all evaluation metrics. The model exhibits remarkable generalization capabilities, particularly in segmenting irregular and boundary-ambiguous regions, while maintaining competitive computational efficiency.
